# The Law Relating to Medical Officers of Health.—I

**Published:** 1903-05-23

**Authors:** J. E. R. Stephens

**Affiliations:** Barrister-at-Law


### HOSPITAL ADMINISTRATION.

### CONSTRUCTION AND ECONOMICS.

### THE LAW RELATING TO MEDICAL OFFICERS OF HEALTH.—I.

By J. E. R. Stephens, Barrister-at-Law.

I.—Appointment of, in London.

Section 106 of the Public Health (London) Act, 1891, provides that every sanitary authority shall appoint one or more medical officers of health for their district. The same person may, with the sanction of the Local Government Board, be appointed medical officer of health for two or more districts by the sanitary authorities of such districts; and the Local Government Board shall prescribe the mode of such appointment and the proportions in which the expenses of such appointment and the salary and charges of such officer shall be borne by such authorities. Every person appointed or reappointed after the commencement of this Act as medical officer of health of a district shall (except during the two months next after the time of his appointment, or except in cases allowed by the Local Government Board) reside in such district or within one mile of the boundary thereof; and if while not so residing as required by this enactment he assumes to act or receives any remuneration as such medical officer of health, he shall cease to hold office. A medical officer of health may exercise any of the powers with which a sanitary inspector is invested. The annual report of a medical officer of health to the sanitary authority shall be appended to the annual report of the sanitary authority. The borough councils are now the sanitary authority for the execution of the Public Health (London) Act, 1891.

Section 108 of the Public Health (London) Act, 1891, provides that, subject to the provisions of this Act as to existing officers, the Local Government Board shall have the same powers as they have in the case of a district medical officer of a poor-law union with regard to the qualification, appointment, duties, salary, and tenure of office of every medical officer of health and sanitary inspector, and one-half of the salary of every such medical officer and sanitary inspector shall be paid by the County Council out of the Exchequer Contribution Account in accordance with section 24 of the Local Government Act, 1888.

A medical officer of health shall be legally qualified for the practice of medicine, surgery, and midwifery, and also either be registered in the Medical Register as the holder of a diploma in sanitary science, public health, or State medicine under section 21 of the Medical Act, 1886; or having been during three consecutive years preceding the year 1892 a medical officer of a district or combination of districts in London or elsewhere with a population according to the last published census of no less than 20,000; or have before the passing of the Local Government Act, 1888, been for not less than three years a medical officer or inspector of the Local Government Board. A medical officer of health shall be removable by the sanitary authority with the consent of the Local Government Board, or by that board, and not otherwise, provided that the Local Government Board shall take into consideration every representation made by the sanitary authority for the removal of any medical officer, whether based on the general interests of the district, on the conduct of such officer, or on any other ground. Any such medical officer shall not be appointed for a limited period only.

A sanitary authority, where occasion requires, may, with the sanction of the Local Government Board, make any temporary arrangement for the performance of all or any of the duties of a medical officer of health or sanitary inspector, and any person appointed by virtue of any such arrangement to perform those duties, or any of them, shall, subject to the terms of his appointment, have all the powers, duties, and liabilities of a medical officer of health or sanitary inspector, as the case may be (section 109).

Article 4 of the Sanitary Officers (London) Order, 1891, issued by the Local Government Board, under section 108 of the Public Health (London) Act, 1891, orders that every medical officer and sanitary inspector appointed under that Act shall be appointed by a majority of those "present and voting." *Quære*, whether that article is not *ultra vires*. By section 2 of the London Government Act, 1899, the quorum of the borough council shall be one-third of the whole number of the council. All questions are to be decided by the majority of the councillors present. A majority of those voting is not sufficient unless it is also a majority of those present. The decision may be determined by a show of hands or by a subsequent decision or poll.

The Sanitary Officers (London) Order, 1891.

The Local Government Board issued the following provisions relating to the appointment and duties of medical officers of health in London.

*Appointment.*—Before any appointment is made under this Order, a statement shall be submitted to us (the Local Government Board) containing the particulars mentioned in the form set forth in the schedule to this Order and such other particulars as may from time to time be required by us (*i.e.*, particulars as to population, etc., salary, etc.). An appointment shall not be made unless an advertisement, specifying the district or part of a district for which such appointment is to be made, together with the amount of salary proposed to be assigned and the day fixed for such appointment, shall have appeared in some public newspaper or newspapers circulating in the district of the sanitary authority at least seven days before the day so fixed. Every officer shall be appointed by a majority of the members present, and voting on the question, at a meeting of the sanitary authority, but such appointment shall be subject to our approval.

*Tenure of Office.*—Every medical officer of health shall continue to hold office until he die, or resign, or be removed by the sanitary authority with our consent, or be removed by us, or be proved to be insane by evidence which we shall deem sufficient. The sanitary authority may, at their discretion, suspend any officer from the discharge of his duties, and shall, in the case of every such suspension, forthwith report the same, together with the cause thereof, to us. The suspension may in the case of a medical officer of health be removed by the sanitary authority or by us; and if the suspension be removed the officer shall forthwith resume the performance of his duties. Where any change in the extent of the area for which any medical officer of health is appointed or in his duties or salary may be deemed necessary, and he shall decline to acquiesce therein, the sanitary authority may, with our consent, but not otherwise, and after six months' notice in writing, signed by their clerk, given to such officer, determine his office. A person shall not be appointed as medical officer of health who does not agree to give one month's notice previous to resigning the office, or to forfeit such sum as may be agreed upon as liquidated damages.

*Salary.*—The sanitary authority shall pay to every officer such salary as may be approved by us, provided always that the sanitary authority may, with our approval, pay to any officer a reasonable compensation on account of extraordinary services, or other unforeseen or special circumstances connected with his duties or the necessities of the district. The salary of every officer shall be payable up to the day on which he ceases to hold office and no longer, subject to any deduction which the sanitary authority may be entitled to make; and in case he shall die while holding such office the proportion of salary (if any) remaining unpaid at his death shall be paid to his personal representatives. But an officer who is suspended, and who without the previous removal of such suspension resigns or is removed, is not entitled to any salary from the date of such suspension. The salary assigned to every officer is payable quarterly on Lady Day, Midsummer Day, Michaelmas Day, and Christmas Day; but the sanitary authority may pay to him at the expiration of every calendar month such proportion as they may think fit on account of the salary to which he would become entitled at the termination of the quarter. All salaries shall be considered as accruing from day to day, and be apportionable in respect of time accordingly.

*Duties of Medical Officer.*—He shall inform himself as far as practicable respecting all influences affecting or threatening to affect injuriously the public health within his district. He shall inquire into and ascertain by such means as are at his disposal the causes, origin, and distribution of diseases within his district, and ascertain to what extent the same have depended on conditions capable of removal or mitigation. He shall by inspection of his district, both systematically at certain periods and at intervals as occasion may require, keep himself informed of the conditions injurious or dangerous to health existing therein. He shall be prepared to advise the sanitary authority on all matters affecting the health of his district, and on all sanitary points involved in the action of the sanitary authority; and in cases requiring it he shall certify, for the guidance of the sanitary authority or of the justices, as to any matter in respect of which the certificate of a medical officer of health or a medical practitioner is required as the basis or in aid of sanitary action. On receiving information of the outbreak of any dangerous infectious disease within his district, he shall visit without delay the spot where the outbreak has occurred, and inquire into the causes and circumstances of such outbreak; and in case he is not satisfied that all due precautions are being taken, he shall advise the persons competent to act as to the measures which may appear to him to be required to prevent the extension of the disease, and shall take such measures for the prevention of disease as he is legally authorised to take under any statute in force in the district, or by any resolution of the sanitary authority. Subject to the instructions of the sanitary authority, he shall direct or superintend the work of the sanitary inspector in the way and to the extent that the sanitary authority shall approve. In any case in which it may appear to him to be necessary or advisable, or in which he shall be so directed by the sanitary authority, he shall himself inspect and examine any animal intended for the food of man which is exposed for sale or deposited in any place for the purpose of sale or of preparation for sale, and any article, whether solid or liquid, intended for the food of man, and sold or exposed for sale, or deposited in any place for the purpose of sale or of preparation for sale. If such animal or article appears to him to be diseased, or unsound, or unwholesome, or unfit for the food of man, he shall seize and carry away the same himself or by an assistant in order to have the same dealt with by a justice according to the provisions of section 47 of the Public Health (London) Act, 1891.

He shall inquire into any offensive process of trade carried on within his district, and report on the appropriate means for the prevention of any nuisance or injury to health therefrom. He shall from time to time inspect any bakehouses which are workshops and are situate within his district, and he shall thereupon report to the sanitary authority whether any steps are necessary to be taken for the purpose of enforcing, as respects such bakehouses, the provisions of the Factory Act.

He shall attend at the office of the sanitary authority or at some other appointed place, at such stated times as they may direct. He shall from time to time report in writing to the sanitary authority his proceedings and the measures which may require to be adopted for the improvement or protection of the public health in his district. He shall in like manner report with respect to the sickness occurring within his district, and the mortality thereof, so far as he is able to ascertain the same. He shall keep a book or books, to be provided by the sanitary authority, in which he shall make an entry of his visits, and notes of his observations and instructions thereon, and also the date and nature of applications made to him, the date and result of the action taken thereon and of any action taken on previous reports; and shall produce such book or books, whenever required, to the sanitary authority.

He shall also make an annual report to the sanitary authority, up to December 31st in each year, comprising a summary of the action taken, or which he has advised the sanitary authority to take during the year, for preventing the spread of disease, and an account of the sanitary state of his district generally at the end of the year. The report shall also contain an account of the inspections and inquiries which he has made as to conditions injurious or dangerous to health existing in his district, and of the proceedings in which he has taken part or advised under any statute, so far as such proceedings relate to those conditions, and also an account of the supervision exercised by him, or on his advice, for sanitary purposes over places and houses that the sanitary authority have power to regulate, with the nature and results of any proceedings which may have been so required and taken in respect of the same during the year. The report shall also record the action taken by him, or on his advice, during the year in regard to offensive trades, to factories and workshops, and to dairies. The report shall also contain tabular statements of the sickness and mortality within his district, classified according to diseases, ages, and localities.

He shall give immediate information to the Local Government Board of any outbreak of dangerous epidemic disease within his district, and he shall transmit to the same authority a copy of each annual report and of any special report made by him.

---

PERMANENT salvage corps, to render aid in case of accident, are about to be organised by the Swiss Alpine Clubs.

THE total cost of the improvements carried out at the Lincoln County Hospital during the last three years is nearly £11,000 Of this amount £4,600 has been promised, £1,000 of which is to be devoted to the erection of a new operating room.

THE wife of a grocer's assistant at Ebbw Vale is reported to have inherited the entire property of a former mistress, consisting of a mansion and estate in the country, a London residence and a considerable sum of money.

